# Unveiling the Roles of Low-Density Lipoprotein Receptor-Related Protein 6 in Intestinal Homeostasis, Regeneration and Oncogenesis

**DOI:** 10.3390/cells10071792

**Published:** 2021-07-15

**Authors:** Jennifer Raisch, Anthony Côté-Biron, Marie-Josée Langlois, Caroline Leblanc, Nathalie Rivard

**Affiliations:** Department of Immunology and Cell Biology, Cancer Research Pavilion, Faculty of Medicine and Health Sciences, Université de Sherbrooke, Sherbrooke, QC J1E 4K8, Canada; jennifer.raisch@usherbrooke.ca (J.R.); Anthony.Cote-Biron@USherbrooke.ca (A.C.-B.); Marie-Josee.Langlois@USherbrooke.ca (M.-J.L.); Caroline.Leblanc2@USherbrooke.ca (C.L.)

**Keywords:** LRP6, intestine, stem cells, regeneration, inflammation, colorectal cancer

## Abstract

Intestinal epithelial self-renewal is tightly regulated by signaling pathways controlling stem cell proliferation, determination and differentiation. In particular, Wnt/β-catenin signaling controls intestinal crypt cell division, survival and maintenance of the stem cell niche. Most colorectal cancers are initiated by mutations activating the Wnt/β-catenin pathway. Wnt signals are transduced through Frizzled receptors and LRP5/LRP6 coreceptors to downregulate GSK3β activity, resulting in increased nuclear β-catenin. Herein, we explored if LRP6 expression is required for maintenance of intestinal homeostasis, regeneration and oncogenesis. Mice with an intestinal epithelial cell-specific deletion of *Lrp6* (*Lrp6*^IEC-KO^) were generated and their phenotype analyzed. No difference in intestinal architecture nor in proliferative and stem cell numbers was found in *Lrp6*^IEC-KO^ mice in comparison to controls. Nevertheless, using ex vivo intestinal organoid cultures, we found that LRP6 expression was critical for crypt cell proliferation and stem cell maintenance. When exposed to dextran sodium sulfate, *Lrp6*^IEC-KO^ mice developed more severe colitis than control mice. However, loss of LRP6 did not affect tumorigenesis in *Apc*^Min/+^ mice nor growth of human colorectal cancer cells. By contrast, *Lrp6* silencing diminished anchorage-independent growth of *BRaf*^V600E^-transformed intestinal epithelial cells (IEC). Thus, LRP6 controls intestinal stem cell functionality and is necessary for BRAF-induced IEC oncogenesis.

## 1. Introduction

Intestinal stem cells, located at the base of intestinal crypts, play a central role in the establishment and maintenance of the intestinal epithelium. These cells undergo asymmetric division leading to migration and differentiation along the intestinal crypt axis [1]. Thus, the intestinal epithelium self-renewal is tightly regulated by interacting intracellular signaling pathways controlling stem and progenitor cell expansion and differentiation. In particular, the Wnt/β-catenin pathway controls proliferation and survival and is required for the maintenance of intestinal stem cells. In the absence of Wnt ligand, β-catenin is destabilized by a destruction complex composed of casein kinase 1 alpha (CK1α), GSK3β, Axin and adenomatous polyposis coli (APC) proteins. β-catenin binds to the destruction complex and is phosphorylated by CK1α and then by GSK3β, which marks β-catenin for ubiquitin-mediated degradation by the proteasome [2]. Wnt, its receptor and co-receptors LRP5/LRP6 form a trimeric complex which recruits the scaffold protein Dishevelled (DVL) and Axin, inducing LRP5/LRP6 aggregation. These aggregates promote phosphorylation of LRP5/LRP6 by CK1γ and GSK3β, releasing β-catenin. β-catenin then accumulates into the cytosol and translocates into the nucleus to interact with nuclear DNA-binding T-cell factor (TCF) and lymphoid enhancer-binding protein (LEF), forming a functional transcription factor promoting target gene expression.

The coreceptors LRP5 and LRP6 are type I single-span transmembrane proteins with a short intracellular domain containing five PPS/TP repeats sites which become phosphorylated upon Wnt activation. In fact, LRP5/6 phosphorylation is necessary for downstream β-catenin stabilization and signaling activity [2]. In this regard, mice lacking the coreceptor LRP6 die at birth, indicating that LRP6 plays a crucial role in tissue development and homeostasis [3,4]. Interestingly, the closely related paralog LRP5 can compensate for the loss of LRP6, as observed during murine intestinal development [5].

Most colorectal cancers (CRC) are initiated by mutations activating the Wnt/β-catenin pathway, commonly in the *APC* gene [6]. Notably, LRP6 expression and phosphorylation are increased in sporadic colorectal tumors, correlating with poor prognosis for patients [7,8,9]. Interestingly, LRP6 phosphorylation and β-catenin transcriptional activity are also both induced by oncogenic *KRAS* or *BRAF* signaling in intestinal epithelial cells (IEC) [7], suggesting that LRP6 phosphorylation may also be involved in colorectal carcinogenesis induced by *KRAS* and *BRAF* oncogenes.

Herein, we analyzed the role of LRP6 in intestinal epithelial homeostasis, inflammation and oncogenesis. For that purpose, we used a conditional knockout approach in mice to eliminate a specific gene (herein LRP6) only in a specific organ (herein intestinal epithelium). Mice are actually very useful in studying such biological processes that have been conserved during the evolution of rodent and primate lineages [10]. Indeed, genomic analyses have revealed important genetic homologies between mouse and human species [11]. Mice with an intestinal epithelial cell-specific deletion of *Lrp6* (*Lrp6*^IEC-KO^) were therefore generated and their intestinal phenotype analyzed. We show that LRP6 is required to maintain the regenerative abilities of intestinal stem cells, thereby protecting the murine epithelium against DSS-induced mucosal damage and inflammation. As expected, LRP6 is dispensable for adenoma formation in *Apc*^Min/+^ mice and for growth of human CRC cells in culture. However, LRP6 is hyperphosphorylated in *KRAS*- and *BRAF*-mutated human CRC cells and important for growth of *BRaf*^V600E^-transformed IEC.

## 2. Materials and Methods

### 2.1. Antibodies and Reagents

Primary antibodies were obtained from the following sources: β-actin (MAB1501R) and phosphorylated ERK1/2 (M8159) from Millipore Sigma (Oakville, ON, Canada), ERK2 (sc-154) from Santa Cruz Biotechnology (Santa Cruz, CA, USA), non-phospho (active) β-catenin (D13A1), LRP5 (D80F2), LRP6 (C5C7), LRP6 (C47E12) and phospho-LRP6 (Ser1490) from Cell Signaling Technology (Danvers, MA, USA) and β-catenin (C14) and BrdU (B44) from BD Biosciences (San Jose, CA, USA). Horseradish peroxidase antibodies were obtained from GE Healthcare Life Sciences (Mississauga, ON, Canada) and alkaline phosphatase-conjugated antibodies from Promega Corporation (Madison, WI, USA). The specific ERK inhibitor SCH772984 was obtained from Cayman Chemical Company (Ann Arbor, MI, USA). Recombinant mouse Wnt3a was obtained from Abcam (Cambridge, MA, USA). Other materials were purchased from Millipore Sigma unless stated otherwise.

### 2.2. Cell Culture

Human CRC cell lines HCT116 (CCL-247, ATCC, Manassas, VI, USA) and HT-29 (HTB-38, ATCC) were maintained in McCoy’s 5A supplemented with 5% FBS (Wisent, Saint-Bruno, QC, Canada). SW48 (CCL-231, ATCC) and Caco-2/15 cell lines (A. Quaroni, Cornell University, Ithaca, NY, USA) were maintained in DMEM (Wisent) containing 10% FBS. IEC6 BRAF^V600E^:ER cells [12] were cultured in DMEM without phenol red supplemented with 5% charcoal-stripped FBS (Wisent).

### 2.3. Mice

C57BL/6 12.4KbVilCre transgenic mice were provided by D. Gumucio (University of Michigan, Ann Arbor, MI, USA). *Lrp6*^loxP/loxP^ and C57BL6/J-*Apc*^Min/+^ mice were purchased from Jackson Laboratory. *Lrp6*^loxP/loxP^; *Villin*-Cre mice (*Lrp6*^IEC-KO^) were compared to *Lrp6*^loxP/+^ or *Lrp6*^loxP/loxP^ control mice. For tumor initiation experiments, *Lrp6*^loxP/loxP^; *Villin*-Cre; *Apc*^Min/+^ mice were compared to *Lrp6*^loxP/+^; *Apc*^Min/+^ or *Lrp6*^loxP/loxP^; *Apc*^Min/+^ control mice. Genomic DNA was extracted from tissue by digestion in 25 mM NaOH/0.2 mM EDTA heated at 95 °C for 1 h followed by addition of an equal volume of 40 mM Tris-HCl (pH 5.5). Genotyping primer sequences and PCR conditions are available upon request. Experiments were approved by the Animal Research Ethics Committee of the Université de Sherbrooke (FMSS-399-18B) in accordance with the Canadian Council on Animal Care standards.

### 2.4. Colitis Induction with DSS and Clinical Evaluation

Ten to twelve-week-old *Lrp6*^IEC-KO^ mice and control littermates were administered dextran sodium sulfate 1.75–2% (DSS, colitis grade; MP Biomedicals, Solon, OH, USA) in drinking water for 7 days. Clinical parameters (weight loss, rectal bleeding and diarrhea) were monitored every day. The disease activity index (DAI) was measured at euthanasia at day 7 [13]. Histological damage scoring was assessed on hematoxylin and eosin-stained longitudinal sections from the entire colon length based on the destruction of normal mucosal architecture, presence and degree of cellular infiltration, extent of muscle thickening, presence or absence of crypt abscesses and the presence or absence of goblet cell depletion, as described in the supplementary Table S1. Clinical scorings were performed in a blinded manner.

### 2.5. Macroadenoma Count, Histological Staining, Immunofluorescence and Immunohistochemistry

Polyps were stained with methylene blue and visualized under an SZ51 stereomicroscope (Olympus, Tokyo, Japan). Polyp sizes were measured with a digital caliper (Thermo Fisher Scientific, Waltham, MA, USA) and polyp numbers were counted from the duodenum to the rectum. For BrdU staining, mice were injected with BrdU (10 μL/g of body weight; Thermo Fisher Scientific) 90 min before euthanasia. Tissues were fixed with paraformaldehyde 4%, paraffin-embedded, sectioned and stained [14]. Immunohistochemistry staining was performed with the EnVision+System Kit (Dako, Burlington, ON, Canada). Slides were visualized with a NanoZoomer slide scanner and NDP.view2 software (Hamamatsu, Boston, MA, USA). Cell counts were performed on well-oriented crypts in a blinded manner.

### 2.6. Enteroids

Crypts were isolated from the jejunum of 8- to 12-week-old mice [15]. Jejunum was opened longitudinally and rinsed with cold PBS. The intestine was then cut into pieces of approximately 5 mm and fragments washed with PBS multiple times by stirring in a 50 mL conical tube. Intestinal fragments were transferred into a new 50 mL conical tube containing 20 mL of 30 mM EDTA in PBS and incubated for 5 min on ice. EDTA was replaced followed by a 20 min incubation on ice with gentle stirring. EDTA was then replaced with 40 mL of PBS and the tube was shaken vigorously until dissociated crypts were visualized in the solution under a microscope. Residual villi were removed by filtering the solution through a 70 μm cell strainer with crypts going through as opposed to residual villi being retained by the filter. Crypts were then centrifuged at 150× *g* for 5 min, crypt pellet was washed twice with 25 mL of Advanced DMEM/F-12 medium, resuspended in phenol red free Matrigel basement membrane (Corning, Glendale, AZ, USA) and plated in a 48-well plate (Corning Costar) (20 μL/well). Enteroids were cultured in Advanced DMEM/F-12 culture medium (Gibco, Waltham, MA, USA) supplemented with 1 mM N-acetylcysteine, 50 ng/mL murine EGF (Life Technologies, Waltham, MA, USA), B27 supplement 1× and N2 supplement 1× (Life Technologies), 10% R-spondin and 10% Noggin-conditioned media. Noggin-Fc and R-spondin 1-Fc were produced in HEK293T cell lines stably expressing R-spondin 1-Fc (C. Kuo, Stanford University, Stanford, CA, USA) or Noggin-Fc (G. R. van den Brink, Hubrecht Institute, Utrecht, The Netherlands). Phase contrast images were taken using a Celldiscoverer 7 live Imaging Station equipped with ZEN software (Carl Zeiss, Toronto, ON, Canada). Enteroid proliferation was evaluated with the Click-it 5-Ethynyl-2′-deoxyuridine (EdU) Alexa Fluor 555 imaging kit (Thermo Fisher Scientific) and visualized with an LSM Olympus FV1000 confocal microscope equipped with FV10-ASW 3.1 software (Olympus; Coopersburg, PA, USA).

### 2.7. Western Blot Analysis

Proteins from scraped intestinal mucosal enrichments were isolated in chilled RIPA buffer. Proteins from cell lines were extracted in chilled Triton X-100 buffer. After a 30 min incubation at 4 °C, samples were sonicated (15%, 10 s). Proteins extracts quantified with the Pierce^TM^ BCA protein assay (Thermo Fisher Scientific) were diluted in Laemmli buffer (62.5 mM Tris-HCl (pH 6.9), 2% sodium dodecyl sulfate, 1% β-mercaptoethanol, 10% glycerol, and 0.04% bromophenol blue) before Western blot analysis.

### 2.8. RNA Extraction and qRT–PCR

RNA was isolated from scraped jejunum mucosa and enteroids with the RNeasy minikit (Qiagen, Toronto, ON, Canada). RT-PCR was performed using the Transcriptor reverse transcriptase with random hexamer primers (Roche Diagnostics, Laval, QC, Canada). Quantitative polymerase chain reaction (qPCR) was performed by the RNomics Platform at Université de Sherbrooke. Target expression was quantified relative to *Pum1*, *Psmc4* and *Tbp* expression. PCR conditions and primer sequences are available upon request.

### 2.9. LRP6 Silencing

LRP6 expression was silenced in human colorectal cancer cell lines with inducible small hairpin RNA (shRNA) plasmids containing 21-mer shRNA sequences targeting the human *LRP6* gene, in the Tet-pLKO-puro plasmid (plasmid #21915, Addgene, Watertown, MA, USA) [16]. A noneffective 21-mer-non-target shRNA plasmid was used as a control [17]. ShRNA expression was induced in stable cell populations with medium containing 50 ng/mL doxycycline. LRP6 expression was silenced in IEC6 BRAF^V600E^:ER cells with small hairpin RNA (shRNA) plasmids containing 29-mer shRNA sequences targeting the rat *Lrp6* gene [7]. A noneffective 29-mer-scrambled shRNA cassette in the pGFP-V-RS vector (OriGene Technologies, Rockville, MD, USA) was used as a control. IEC6 BRAF^V600E^:ER cells were infected with control or *Lrp6*-shRNA lentiviruses and selected with puromycin (1 μg/mL).

### 2.10. Soft Agar Assay

Inducible shRNA cell lines were treated with 50 ng/mL doxycycline 48 h before seeding. Cells were counted using a hemocytometer and 30,000 (SW48) or 10,000 cells (HCT116) were seeded in 0.7% agarose type VII [18] and cultured in media containing 50 ng/mL doxycycline and 10% FBS. Untransfected or shRNA-expressing IEC6 BRAF^V600E^:ER cells were treated with 250 nM 4OH-Tamoxifen (Cayman Chemical) 16 h before seeding 30,000 cells per well in 0.7% agarose type VII and continuous culturing in media containing 250 nM 4OH-Tamoxifen. Cells were grown for 14 (HCT116), 21 (SW48) or 21 to 35 days for IEC6 BRAF^V600E^:ER cells. Colonies were stained with 0.5 mg/mL MTT (3-(4,5-dimethylthiazol-2-yl)-2,5-diphenyltetrazolium bromide) added to wells for 4 h at 37 °C. Images were acquired using an Infinity VX2 1100/26MX Imaging System (Vilber Lourmat, Marne-la-Vallée, France). Colony number and size were assessed using ImageJ software (National Institutes of Health, Bethesda, MD, USA).

### 2.11. Clonogenic Assay

Inducible shRNA cell lines were treated with 50 ng/mL doxycycline 48 h before seeding. Approximately 1000 cells were seeded into six-well plates and cultured in media containing 50 ng/mL doxycycline for 14 days. Colonies were then stained with a PBS solution containing 0.05% crystal violet, 1% formaldehyde and 1% methanol. Phase contrast images were taken with a Zeiss Cell Discoverer 7 live Imaging Station equipped with ZEN software. Colonies were counted using ImageJ software.

### 2.12. Statistical Analyses

Assays were carried out at least in triplicate. Results are representative of three independent experiments if not stated otherwise. Data were analyzed with Student’s *t*-test for populations following a normal curve or Mann–Whitney U test in other cases. Results were considered statistically significant at *p* ≤ 0.05. Graphs and statistics were generated using GraphPad Prism software (Prism, San Diego, CA, USA).

## 3. Results

### 3.1. Lrp6 Deletion Does Not Alter Intestinal Architecture and Homeostasis but Impairs Wnt Target Gene Expression

**Figure 1:**
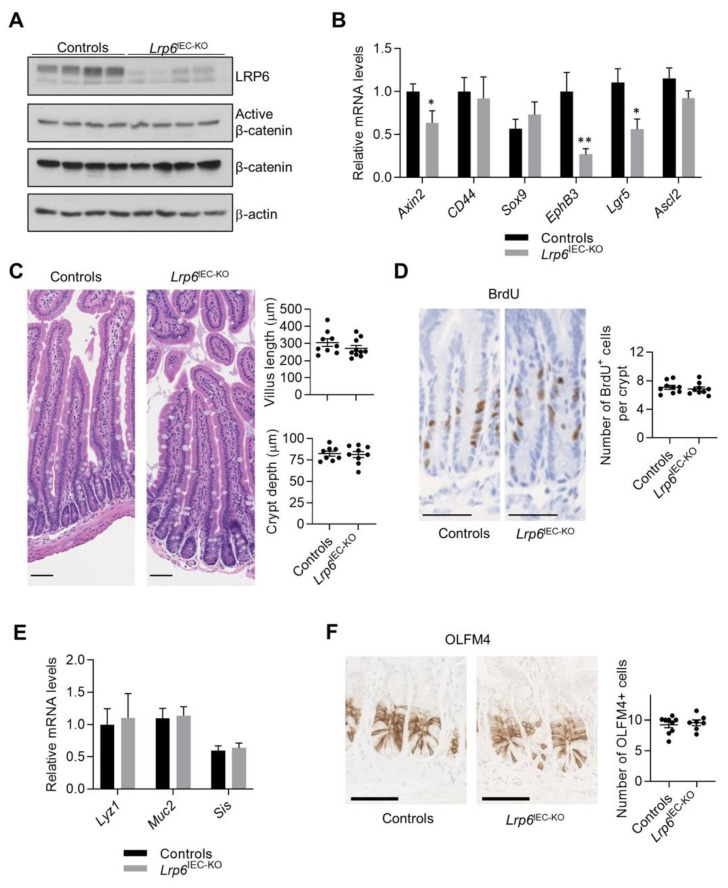
Loss of LRP6 expression affects Wnt target gene expression without altering intestinal architecture. (**A**) LRP6 and β-catenin expression was analyzed in 12-week-old murine small IEC-enriched lysates by Western blot. (**B**) β-catenin target gene expression was evaluated by qPCR of IEC-enriched RNA (*n* ≥ 5). (**C**) Villus length and crypt depth were determined on H&E-stained jejunal sections (*n* ≥ 8). Bars: 100 µm. (**D**) Proliferation was assessed after BrdU immunohistochemistry on mutant and control jejunal sections (*n* = 9). Bars: 50 µm. (**E**) The expression of mucin 2 (*Muc2*), lysozyme (*Lyz1*) and sucrase-isomaltase (*Sis*), the main markers of goblet, Paneth and absorptive cells, respectively, was evaluated by qPCR on IEC-enriched RNA (*n* ≥ 8). (**F**) OLFM4 immunohistochemistry was performed on 12-week-old control and *Lrp6^I^*^EC-KO^ jejunal sections (*n* ≥ 7) to visualize intestinal stem cells. Bars: 50 µm. Data are expressed as mean ± SEM. Mann–Whitney U test * *p* ≤ 0.05, ** *p* ≤ 0.01.

To address the importance of LRP6 in intestinal development and architecture, we bred *Lrp6*^loxP/loxP^ mice with *Villin*-Cre mice that express the Cre recombinase in intestinal epithelial cells [19] leading to specific loss of LRP6 expression in these cells (Figure 1A). Because LRP6 is a co-receptor involved in the activation of the Wnt/β-catenin signaling pathway, we verified the activation status of this pathway by analyzing the levels of total and activated β-catenin protein. As shown in Figure 1A, total and active β-catenin levels were not significantly different in mutant and control mucosae. Although we did not observe a difference in β-catenin expression levels, we cannot exclude that subtle changes might occur in its transcriptional activity. We therefore analyzed the expression levels of Wnt/β-catenin target genes. Intriguingly, while *Cd44*, *Sox9* and *Ascl2* gene expression remained unaffected by loss of epithelial LRP6, *Axin2*, *Lgr5* and *EphB3* gene expression was significantly decreased in *Lrp6*^IEC-KO^ mucosal enrichments (Figure 1B). Histological analyses of *Lrp6*^IEC-KO^ mouse jejunal sections revealed, however, no difference in intestinal architecture, villus length, crypt depth and proliferation in comparison to control littermates (Figure 1C,D). No histological change was observed in the colonic epithelium as well (Figure S1A,B). Expression levels of genes encoding mucin 2, lysozyme and sucrase-isomaltase, the main markers of goblet, Paneth and absorptive differentiated cells, respectively, were also comparable between control and mutant mice (Figure 1E). The decreased expression of *Lgr5* in *Lrp6* KO mucosal enrichments suggests a change in the number of active cycling stem cells, also named crypt base columnar (CBC) cells. We therefore performed immunohistochemistry against OLFM4, a robust marker for these cells. As shown in Figure 1F, similar numbers of OLFM4-positive cells were found in *Lrp6*^IEC-KO^ and control mice. Thus, while loss of LRP6 in intestinal epithelial cells reduced the expression of some Wnt target genes, including the stem cell marker *Lgr5*, it was not sufficient to perturb homeostatic maintenance of the intestinal epithelium under normal conditions.

### 3.2. LRP6 Expression Is Required for Ex Vivo Intestinal Crypt Regeneration

**Figure 2:**
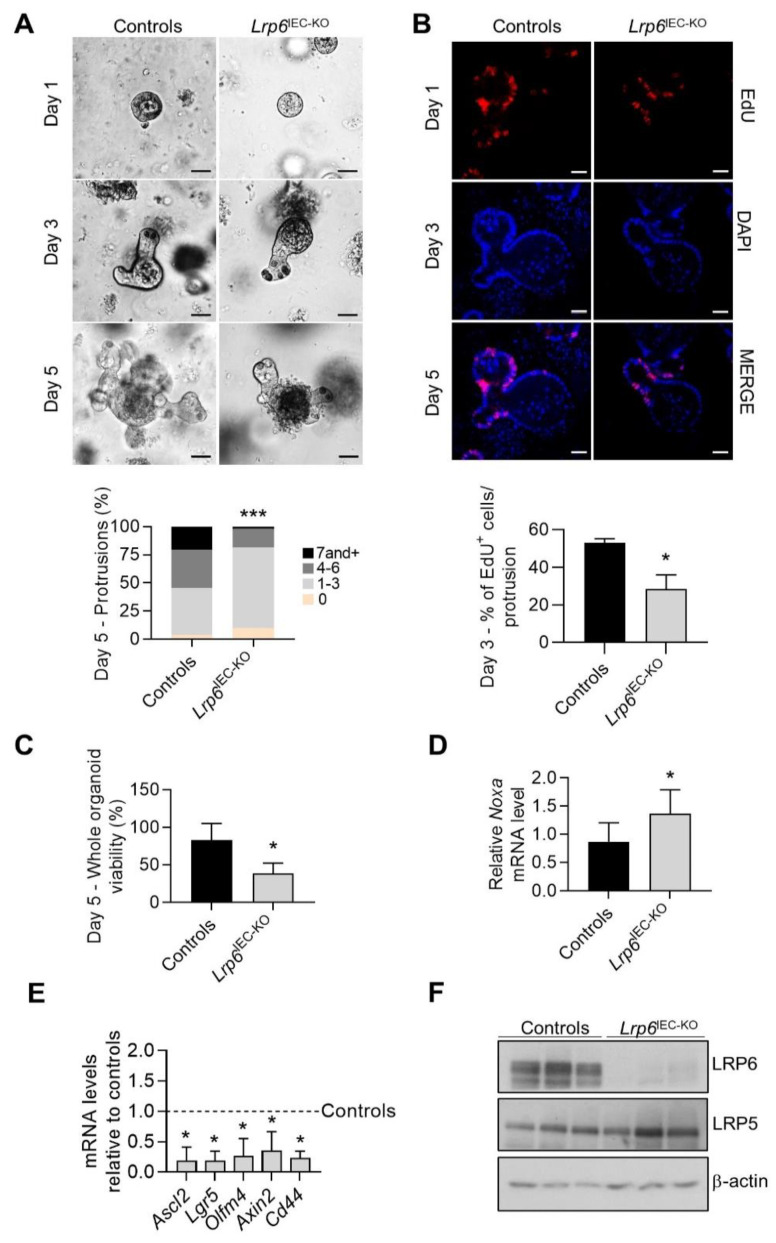
Loss of LRP6 expression impairs enteroid development. (**A**) Phase contrast images of enteroids 1, 3 and 5 days after seeding. Bars: 50 µm. Protrusions per enteroid were counted 5 days after seeding. The average of 6 independent experiments, each with at least 40 organoids, is summarized in the graph. Chi-square, *** *p* ≤ 0.001. (**B**) Enteroid proliferation was evaluated 3 days after seeding by EdU incorporation and immunofluorescence to evaluate the ratio of proliferative cells per protrusion. The average of 4 independent experiments, each with at least 8 protrusions, is summarized in the graph. Bars: 50 µm. Unpaired *t*-test, * *p* ≤ 0.05. (**C**) Enteroid viability was determined 5 days after seeding by phase contrast microscopy. The average of 3 independent experiments, each with at least 50 organoids, is summarized in the graph. Unpaired *t*-test, * *p* ≤ 0.05. (**D**,**E**) *Noxa*, *Ascl2*, *Lgr5*, *Axin2* and *Cd44* expression was evaluated by qPCR by comparing mutant and control enteroid RNA isolated 3 days after seeding (*n* ≥ 3). Unpaired *t*-test, * *p* ≤ 0.05. (**F**) Enteroid LRP6 and LRP5 expression was analyzed by Western blot 3 days after seeding. Data are expressed as mean ± SD.

Since the expression of some Wnt target genes is reduced upon *Lrp6* deletion, we next examined the regenerative abilities of intestinal stem cells by preparing enteroids from control and *Lrp6*^IEC-KO^ mice. Notably, development of *Lrp6*^IEC-KO^ enteroids was compromised in comparison to control enteroids. Indeed, the number of protrusions was significantly reduced in enteroids lacking *Lrp6* expression (Figure 2A). Three days after seeding, proliferation of *Lrp6*-deficient enteroids was significantly decreased in comparison to control enteroids, as visualized by a reduced number of EdU-positive cells (Figure 2B). After 5 days, *Lrp6*-deficient enteroids degenerated and died (Figure 2C) and increased expression of the pro-apoptotic *Noxa* gene was noticed (Figure 2D). To examine the effect of *Lrp6* deletion on Wnt signaling in enteroids, we determined the mRNA expression level of Wnt target stem cell genes by quantitative RT-PCR. There was a significant reduction in the expression of *Axin2*, *Lgr5*, *Ascl2* and *Cd44* in *Lrp6*^IEC-KO^ enteroids, 3 days after seeding (Figure 2E). Since LRP5 expression was also detected in enteroids (Figure 2F), we tested the ability of exogenous Wnt3a to rescue the growth defect observed in *Lrp6*^IEC-KO^ enteroids. As shown in Figure S2A,B, *Lrp6*-deficient enteroids still degenerated and died despite the addition of Wnt3a to culture medium. These results suggest that LRP6 is much more important than LRP5 for the control of intestinal stem cell renewal and crypt regeneration, at least ex vivo.

### 3.3. Lrp6^IEC-KO^ Mice Are More Sensitive to DSS-Induced Epithelial Damage and Colitis

**Figure 3:**
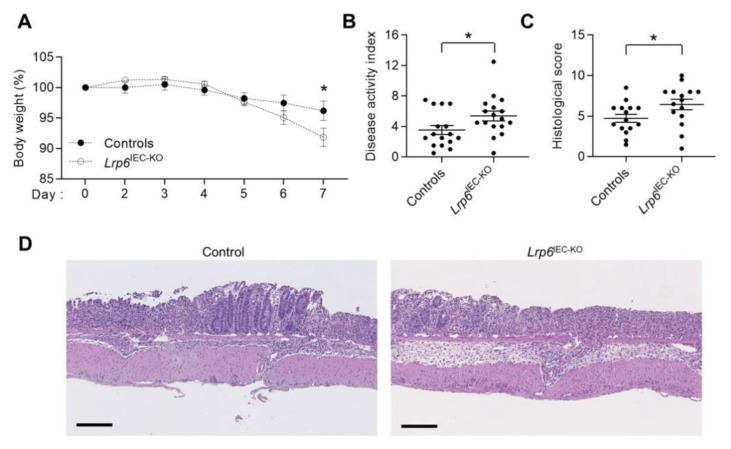
*Lrp6*^IEC-KO^ mice are more sensitive to DSS-induced colitis. Ten- to twelve-week-old *Lrp6*^IEC-KO^ and control mice were treated with 1.75–2% DSS in water for 7 days. (**A**) Body weight was measured every day (*n* = 18). (**B**) Disease activity index of *Lrp6*^IEC-KO^ and control mice was calculated by scoring stool softness, occult fecal blood, rectal bleeding and colon rigidity at euthanasia (*n* ≥ 15). (**C**,**D**) H&E staining was performed on *Lrp6*^IEC-KO^ and control colon tissues to score damage (**C**) according to the extent of mucosal architecture destruction, immune cell infiltration, goblet cell depletion, muscle thickening and crypt abscesses (*n* ≥ 15). Bars: 200 µm. Data are expressed as mean ± SEM. Mann–Whitney U test, * *p* ≤ 0.05.

We next evaluated the susceptibility of control and *Lrp6*^IEC-KO^ mice to develop colonic inflammation in response to dextran sodium sulfate (DSS) administration. As shown in Figure 3A,B, DSS administration induced acute colitis in control mice after 7 days, as shown by significant weight loss, diarrhea and occasional bleeding, resulting in a relatively high Disease Activity Index (DAI). Interestingly, *Lrp6*^IEC-KO^ mice exhibited more weight loss and significantly higher DAI associated with more severe diarrhea, rectal bleeding and bloody stools. Hematoxylin and eosin staining was also performed on colonic tissue to score damage according to the extent of mucosal architecture destruction, the presence of cellular infiltration, crypt abscesses, goblet cell depletion and the extent of muscle thickening; typical alterations observed during colitis. As shown in Figure 3C,D, *Lrp6*^IEC-KO^ mice exhibited a significantly higher histological score than control mice, indicating that *Lrp6*^IEC-KO^ intestinal mucosa was more affected following DSS treatment.

### 3.4. LRP6 Is Dispensable for Adenoma Formation in Apc^Min/+^ Mice and for Growth of Human Colorectal Cancer Cells

**Figure 4:**
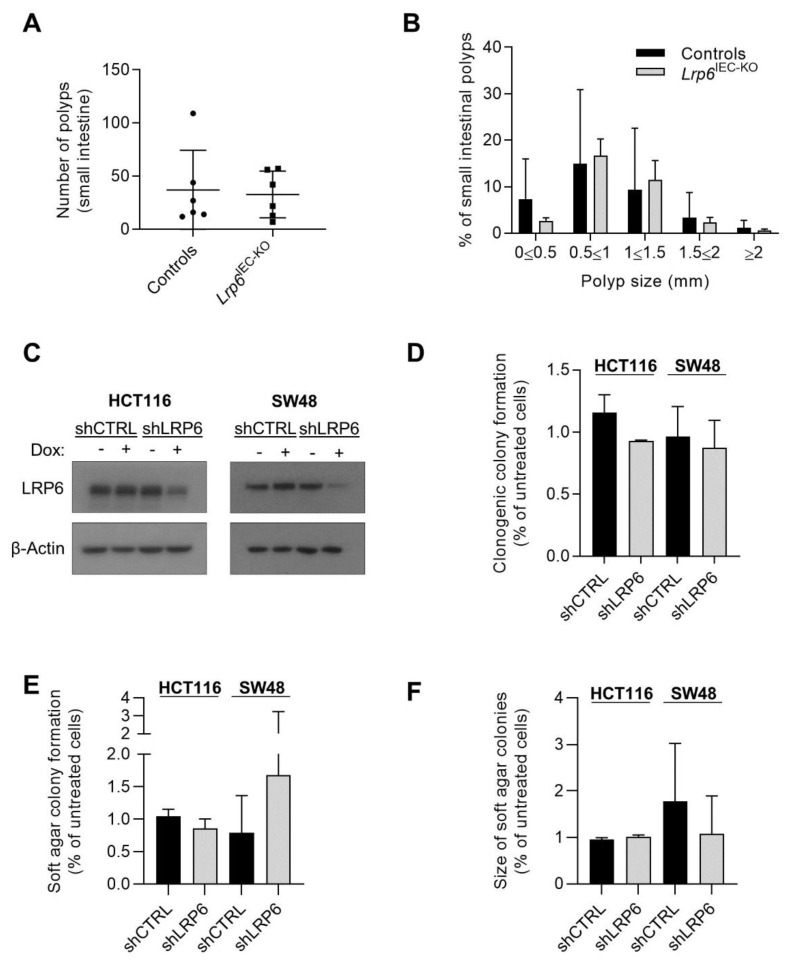
Role of LRP6 in adenoma formation and growth of colorectal cancer cells. (**A**) Polyp number was counted in 12-week-old *Lrp6*^loxP/loxP^; *Villin*-Cre; *Apc*^Min/+^ small intestines compared to controls (*Lrp6*^loxP/+^; *Apc*^Min/+^ or *Lrp6*^loxP/loxP^; *Apc*^Min/+^) (*n* ≥ 5). (**B**) Small intestinal polyp size (diameter) was analyzed in *Lrp6*^loxP/loxP^; *Villin*-Cre; *Apc*^Min/+^ and control mice (*n* ≥ 5). (**C**) Expression of a shRNA against *LRP6* (shLRP6) or a non-target shRNA (shCTRL) was induced with 50 ng/mL doxycycline for 48 h in HCT116 and SW48 cells. Loss of LRP6 expression was determined by Western blot. (**D**) shLRP6 or shCTRL expression was induced with 50 ng/mL doxycycline 48 h before seeding cells at low density for clonogenic assays. Cells grown for 14 days were stained with crystal violet and colonies counted. Three independent experiments are summarized in the graph. (**E**,**F**) shLRP6 or shCTRL expression was induced with 50 ng/mL doxycycline 48 h before soft agar seeding. HCT116 cells were grown for 14 days and SW48 cells for 21 days in soft agar before MTT staining. The number of colonies (**E**) and their size (**F**) were evaluated. Three independent experiments are summarized in the graph. Data are expressed as mean ± SD. Paired *t*-test.

Because initiation of most colorectal cancers (CRC) is often due to activating mutations in Wnt/β-catenin signaling, we verified the role of LRP6 in *Apc*^Min/+^ mice, which are heterozygous for an *Apc* mutation frequently found in human CRC, and which spontaneously develop intestinal adenomas [20]. *Lrp6*^IEC-KO^ mice were crossed with *Apc*^Min/+^ mice and intestinal tumor load was analyzed after 3 months. Unexpectedly, the absence of LRP6 did not significantly affect tumor development in *Apc*^Min/+^ mice (Figure 4A,B). We next assessed the role of LRP6 in two established human CRC cell lines exhibiting activated Wnt signaling, namely HCT116 (*CTNNB1* mutation) and SW48 (*APC* mutation) cells, by using a doxycycline-inducible shRNA expression vector targeting *LRP6* mRNA, to knockdown LRP6 protein expression. LRP6 knockdown (Figure 4C) was not sufficient to alter clonogenic potential nor anchorage-independent growth of HCT116 and SW48 cancer cells (Figure 4D–F).

### 3.5. ERK-Dependent Phosphorylation of LRP6 in KRAS- and BRAF-Mutated Colorectal Cancer Cells—A Role for LRP6 in BRAF-Induced Intestinal Oncogenesis

**Figure 5:**
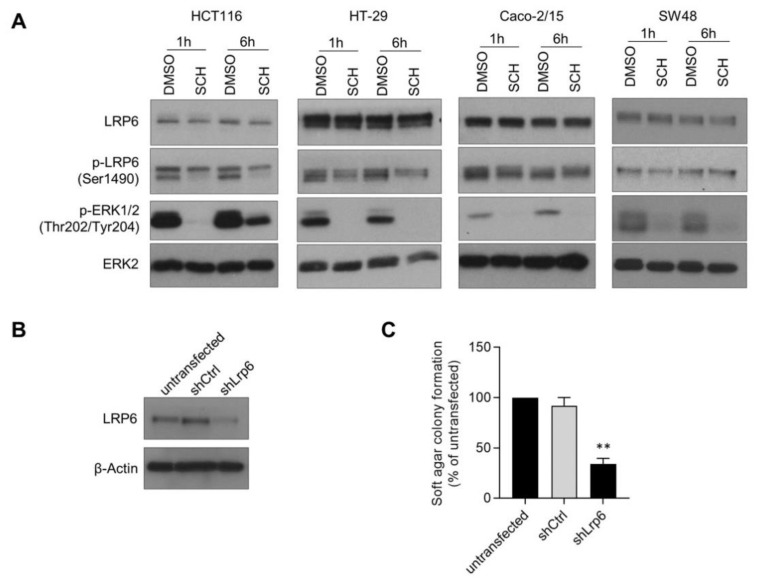
LRP6 phosphorylation contributes to BRAF-induced intestinal oncogenesis. (**A**) *KRAS*-mutated HCT116, *BRAF*-mutated HT-29, *KRAS* and *BRAF* wild-type Caco-2/15 and SW48 cells were treated with SCH772984 (1 µM) or DMSO for 1 and 6 h. Protein expression and phosphorylation were analyzed by Western blot. (**B**) Loss of LRP6 expression was determined by Western blot in IEC-6 cells expressing the inducible BRAF^V600E^:ER fusion protein, and shRNA against *Lrp6* (shLrp6) or a control shRNA (shCtrl) and compared to non-transfected cells. (**C**) IEC6 BRAF^V600E^:ER cells stably expressing shLrp6, shCtrl or that were non-transfected were treated with 250 nM 4OH-tamoxifen 16 h before soft agar seeding. Cells were grown for 3 to 5 weeks in soft agar before MTT staining and colony counting. Three independent experiments are summarized in the graph. Data are expressed as mean ± SD. Paired *t*-test, ** *p* ≤ 0.01.

Previously, we reported that oncogenic KRAS and BRAF signaling in rodent intestinal epithelial cells (IEC-6 cells) activates the Wnt/β-catenin pathway by increasing LRP6 phosphorylation [7]. Accordingly, treatment of *KRAS* (HCT116) and *BRAF* (HT-29)-mutated human CRC cells with the ERK1/2 inhibitor, SCH7729846, reduced LRP6 phosphorylation on serine 1490, a residue required for Wnt signal transduction. Interestingly, LRP6 phosphorylation was not or only barely affected by the SCH7729846 inhibitor in *KRAS* and *BRAF* wild-type CRC cells (Caco-215 or SW48) (Figure 5A). These data suggest that LRP6 phosphorylation may be instrumental for oncogenesis induced by abnormal ERK signaling. We therefore knocked down LRP6 expression in IEC-6 cells transformed by the 4-hydroxytamoxifen-inducible BRAF^V600E^:ER fusion protein (Figure 5B). As shown in Figure 5C, LRP6 silencing clearly reduced the ability of oncogenic BRAF to induce anchorage-independent growth in IEC-6 cells.

## 4. Discussion

Although LRP6 is involved in Wnt/β-catenin signal transduction, its role in the maintenance of intestinal homeostasis remains unclear [5]. Herein, we demonstrated that IEC-specific *Lrp6* deletion in mice does not affect intestinal development nor crypt cell proliferation and differentiation. Because the maintenance of Wnt signaling in the intestinal epithelium is absolutely required to maintain homeostasis, total and active β-catenin expression was analyzed as a readout of Wnt pathway activation. Levels of total and active β-catenin proteins remained similar between control and *Lrp6*-deficient mucosae. Nonetheless, expression of some Wnt target genes, namely *Lgr5*, *Axin2* and *Ephb3*, was decreased in *Lrp6*^IEC-KO^ mice, suggesting that intestinal crypt cells might be somehow less responsive to Wnt stimulation in the absence of the LRP6 protein. One of the reduced genes, *Lgr5*, was previously recognized as a marker of active cycling stem cells, located at the crypt base of the crypt–villus axis, and whose self-renewal is dependent on Wnt signaling. Hence, impaired activation of Wnt pathway activation may result in decreased LGR5-positive active stem cell numbers, as observed after β-catenin deletion in the murine intestinal epithelium [21]. However, immunohistochemistry against OLFM4 protein, a robust marker for these stem cells [1], indicates that *Lrp6*^IEC-KO^ and control crypts retain similar numbers of active stem cells. Additionally, expression of *Ascl2*, a Wnt-responsive master transcription factor that controls LGR5-positive intestinal stem cell gene expression programs [22], remains unaffected without LRP6 in the murine intestinal epithelium. Thus, although *Lgr5* gene expression is reduced, the number of actively cycling stem cells and proliferative cells in *Lrp6*-deficient intestinal crypts remain comparable to what is observed in control crypts. This could be at least explained by the fact that LRP5, a LRP6 ortholog, is also expressed in murine crypt epithelial cells. Indeed, LRP5 and LRP6 can play compensatory functions as reported during murine intestinal epithelial development [5]. A modest increase in LRP5 protein expression was observed in *Lrp6*^IEC-KO^ intestines in comparison to controls (Figure S3A). This slight LRP5 increase could be sufficient to sustain enough Wnt signaling and maintain intestinal homeostasis and renewal in mice. Alternatively, additional factors such as fibroblast growth factors (FGF2 and FGF9) [23], glucagon-like pertide-2 [24], heparin-binding epidermal growth factor-like growth factor [25] and epidermal growth factor (EGF) [26] have been also shown to stimulate β-catenin signaling in intestinal crypts and thus, could compensate for the absence of LRP6 for the maintenance of intestinal homeostasis.

Interestingly, *Lrp6*^IEC-KO^ jejunal crypt enteroids showed hindered proliferation and died a few days after seeding, probably by increased apoptosis, as suggested by the increased expression of the pro-apoptotic gene *Noxa*. Decreased *Lrp6*-deficient enteroid proliferation and viability may be due to Wnt signaling inhibition in stem and progenitor cells, as revealed by the substantially decreased expression of the Wnt target genes *Lgr5*, *Ascl2*, *Axin2* and *Cd44*. Additionally, *Olfm4* was also significantly decreased in *Lrp6*-deficient enteroids. Decreased expression of *Olfm4*, *Ascl2* and *Lgr5* strongly indicates that functionality of active stem cells in enteroids was impaired in the absence of LRP6. Since such alterations were not observed in vivo, one could speculate that underlying mesenchymal niche cells likely maintain stem cell homeostasis in *Lrp6*^IEC-KO^ mice. Moreover, our findings are reminiscent of those observed with *Math1*- and *Wnt3*-inducible-IEC-KO mouse models. While no alteration in stem cell numbers and Wnt signaling, as assessed by measuring active β-catenin levels, was observed in vivo, enteroids derived from these KO mice could not grow [27,28]. Of note, MATH1 is an essential driver of differentiation of secretory cells, including Paneth cells, which produce growth factors such as EGF, Notch and Wnt3, which collectively promote the growth and maintenance of LGR5-positive stem cells. Therefore, the absence of MATH1, specifically in intestinal epithelial cells, leads to crypt enteroid growth arrest, resulting from the inhibition of Paneth cell differentiation and of the production of growth factors, especially Wnt3 [28]. Thus, these studies have revealed that Paneth cell-secreted Wnt3 is not the only niche factor involved in stem cell maintenance in vivo, suggesting that redundant Wnt signals exist in mice to ensure intestinal homeostasis. Indeed, intestinal stromal cells act as important regulators of the stem cell niche, by producing and secreting several Wnt factors, notably Wnt2b and Wnt4 [29,30,31,32,33]. Although *Wnt2b* or *Wnt4* gene expression was not modulated in *Lrp6*^IEC-KO^ intestines (Figure S3B), other mesenchymal-derived growth factors may contribute to the maintenance of intestinal homeostasis in *Lrp6*^IEC-KO^ mice, as described above. That being said, *Lrp6*^IEC-KO^ enteroids continued to degenerate and die despite the addition of Wnt3a (Figure S2), indicating that LRP6, but not LRP5, is required for Wnt signaling transduction in intestinal crypt cells. Likewise, LRP6 has been reported to be much more potent than LRP5 in activating Wnt signaling in other cell types [34,35].

A variant of the human *LRP6* gene was recently found to be frequently expressed in early onset ileal Crohn’s Disease, a chronic intestinal inflammatory disorder [36]. We therefore analyzed the sensitivity of *Lrp6*^IEC-KO^ mice to intestinal inflammation. Even though no difference in *Lrp6*^IEC-KO^ colons was observed compared to controls in basal conditions (Figure S1A–C), DSS-treated *Lrp6*^IEC-KO^ mice displayed increased DAI and more severe mucosal histological damage than control mice. The mechanisms explaining this increased susceptibility to colitis in the absence of *Lrp6* still need to be determined. However, although LRP6 was dispensable for the maintenance of intestinal homeostasis under homeostatic conditions, LRP6 may be important to ensure adequate intestinal mucosal recovery through crypt regeneration, following epithelial damage. Indeed, histological analysis of *Lrp6*-deficient colonic mucosae after DSS treatment revealed the presence of large areas still denuded of epithelium, as opposed to DSS-treated control mice. Increased epithelial cell proliferation is necessary to allow intestinal mucosal recovery after DSS-induced injury [37]. In this regard, IBD patient mucosa exhibits enhanced epithelial proliferation, which decreases during resolution of inflammation [38]. Altogether, these data suggest that, by promoting crypt regeneration, LRP6 may protect the intestinal epithelium against damage and inflammation.

Most CRC develop via adenomatous polyps initiated by mutations activating the Wnt/β-catenin pathway, commonly in the adenomatous polyposis coli (*APC*) gene, or less frequently in the β-catenin (*CTNNB1*) gene. Interestingly, LRP6 overexpression in CRC cells activates Wnt/β-catenin signaling and cell migration [9]. However, whether LRP6 expression contributes to Wnt signaling and CRC tumoral properties, especially in CRC with *APC* or β-catenin mutations, remains unclear. Chen and He (2019) did not observe changes in Wnt signaling activation in APC-deficient CRC cells after *Lrp6* knockout by CRISPR/Cas9 genome editing [39]. In contrast, Saito-Diaz et al. (2018) demonstrated that LRP6 knockdown in CRC cells, even those exhibiting *APC* mutations, significantly reduced Wnt signaling [40] and this was recently confirmed by single cell analyses of endogenous β-catenin levels [41]. Unfortunately, in their study, Saito-Diaz et al. (2018) did not analyze the impact of LRP6 knockdown on CRC cell growth. Herein, we show that significantly decreased expression of LRP6 did not affect CRC cell clonogenic potential nor ability to form colonies in soft agar. Of note, the two cell lines analyzed, namely HCT116 and SW48 cells, exhibit mutations in *CTNNB1* (β-catenin) and *APC* genes, respectively. Thus, our results suggest that CRC cells harboring activating mutations in Wnt signaling do not need LRP6 expression for their growth. Likewise, epithelial *Lrp6* deletion does not affect intestinal tumor load and size in *Apc*^Min/+^ mice. Taken together, these findings support the notion that epithelial LRP6 expression is dispensable not only for the maintenance of intestinal homeostasis but also for tumorigenesis induced by aberrant β-catenin signaling.

Serrated adenocarcinomas, which account for 20–30% of CRC, follow an alternative pathway independent of *APC* mutations, in which serrated polyps replace traditional adenoma as the CRC precursor lesion [42]. This pathway involves early *BRAF* mutations, excess CpG island methylation and DNA microsatellite instability (MSI). Previous reports have demonstrated that the expression of constitutively active mutants of BRAF or KRAS in non-transformed IEC, such as IEC-6 cells, is sufficient to promote tumoral transformation [7,43]. We previously reported that expression of *BRAF*^V600E^ or *KRAS*^G12D^ oncogenes in IEC activates β-catenin nuclear transcriptional activity and target gene expression [7]. Notably, LRP6 was phosphorylated on serine-1490 and threonine-1572 in a MEK-dependent manner, in IEC transformed by oncogenic KRAS or BRAF, thus providing a mechanism integrating KRAS/MAPK and canonical Wnt/β-catenin signaling during intestinal transformation. Indeed, both the serine-1490 and threonine-1572 residues are localized within the PPPS/TP motifs of the LRP6 co-receptor, motifs required for Wnt signal transduction. Herein, we observed ERK-dependent LRP6 phosphorylation in human CRC cell lines with *KRAS* or *BRAF* mutations. This suggests that LRP6 phosphorylation is involved in oncogenic signaling induced by *KRAS* or *BRAF* mutations. However, LRP6 silencing in HCT116 cells which harbored a *KRAS* mutation did not significantly alter anchorage-independent growth nor clonogenic potential. It is important to note that, in addition to *KRAS* mutation, HCT116 cells exhibit several other genetic alterations including *CTTNB1* mutation, *PIK3CA* mutation, CIMP and microsatellite instability that may also stimulate their growth. To circumvent this situation, we used a simpler model, namely IEC-6 stably expressing an inducible oncogenic form of human BRAF, BRAF^V600E^:ER. Following stimulation with 4-hydroxytamoxifen, BRAF^V600E^ induced ERK1/2 signaling, cell transformation and anchorage-independent growth [43]. LRP6 knockdown in this model significantly reduced the capacity of BRAF^V600E^ to induce colony formation in soft agar, which is a measure of anchorage-independent growth potential correlating with tumorigenic growth in vivo. Nonetheless, it will be important in future studies to confirm this function of LRP6 in a more relevant model of human cancer such as patient-derived CRC organoids (e.g., with or without *BRAF* mutation). Indeed, fewer genetic or epigenetic events are required to induce a malignant transformation in murine cells compared with human cells [10], and patient-derived tumoroids, which grow as irregular compact structures, maintain hallmarks of the isolated CRC tumor type [44]. They thus represent a more relevant model for drug screening in cancer [45].

## 5. Conclusions

In conclusion, although LRP6 does not play an essential role in the regulation of intestinal homeostasis and *Apc*-induced tumorigenesis in mice, LRP6 promotes crypt regeneration after epithelium damage and contributes to oncogenesis induced by *BRAF* oncogene. Our results suggest that LRP6 could represent a novel target for colorectal tumorigenesis associated with *KRAS* or *BRAF* mutations.

## Data Availability

No new data were created or analyzed in this study. Data sharing is not applicable to this article.

## Supplementary Materials

The following are available online at https://www.mdpi.com/article/10.3390/cells10071792/s1. Table S1: Histological damage scoring description, Figure S1: Loss of LRP6 expression does not alter colonic architecture. (A) Crypt depth was determined on H&E-stained colonic sections from *Lrp6*^IEC-KO^ mice and control littermates (*n* ≥ 5). Scale bars, 50 µm. (B) Proliferative cells were observed by BrdU immunohistochemistry on colonic sections from mutant mice compared with control littermates (*n* = 6). Scale bars, 50 µm. (C) Colon weight (mg), colon length (cm) and body weight (g) were measured in 3-month-old mice (*n* ≥ 5). Data are expressed as mean ± SEM. Mann–Whitney U test, Figure S2: Wnt3a does not rescue impaired *Lrp6*^IEC-KO^ enteroid development. (A) Enteroid viability was determined 5 days after seeding by phase contrast microscopy on *Lrp6*^IEC-KO^ organoids treated or not with Wnt3a (100 ng/mL) from day 1 to day 5. The average of 3 independent experiments, each with at least 50 organoids, is summarized in the graph. Unpaired *t*-test. (B) Protrusions per enteroid were counted 5 days after seeding on *Lrp6*^IEC-KO^ organoids treated or not with Wnt3a (100 ng/mL) from day 1 to day 5. The average of 3 independent experiments, each with at least 40 organoids, is summarized in the graph. Chi-square test. Data are expressed as mean ± SD, Figure S3: Loss of LRP6 expression impacts on other proteins implicated in the Wnt/β-catenin pathway. (A) LRP5 expression was determined by Western blot on small intestinal epithelial cell-enriched lysates from 12-week-old *Lrp6* mutant and control mice. (B) Relative expression level of different Wnt factors was evaluated by qPCR from small intestinal lysate RNA of mutant mice compared to littermate controls. Data are expressed as mean ± SD. Mann–Whitney U test.
