# Supplementary material for: Unveiling the Roles of Low-Density Lipoprotein Receptor-Related Protein 6 in Intestinal Homeostasis, Regeneration and Oncogenesis
Source: Cells. 2021 Jul 15;10(7):1792. doi: 10.3390/cells10071792 (PMC8307932; doi:10.3390/cells10071792)
Supplement: Supplementary file 1 [file cells-10-01792-s001.zip › cells-1254428-supplementary/RaischTableS1.pdf]

Table S1. Histological damage scoring

Histological features	Score	Observations
Extent of destruction of normal mucosal architecture	0	Normal
	1	Mild (destruction in less than 25% of the colon length)
	2	Moderate
	3	Extensive damage (destruction in more than 50% of the colon length)
Presence and degree of cellular infiltration	0	Normal
	1	Few and small areas of immune cell infiltrates
	2	Moderate number and/or size of immune cell infiltrates
	3	Transmural infiltration
Extent of muscle thickening	0	Normal
	1	Mild (1/3 thickening maximum in some areas)
	2	Moderate (2/3 thickening maximum in some areas)
	3	Extensive thickening
Presence or absence of crypt abscesses	0	Absent
	1	Present
Presence or absence of goblet cell depletion (at least 25% reduction in some inflamed areas)	0	Absent
	1	Present

Histological damage scoring was assessed on hematoxylin and eosin-stained sections of the entire colon length. All scores were added for a maximum score of 11.
