# Supplementary figures and images for: Unveiling the Roles of Low-Density Lipoprotein Receptor-Related Protein 6 in Intestinal Homeostasis, Regeneration and Oncogenesis
Source: Cells. 2021 Jul 15;10(7):1792. doi: 10.3390/cells10071792 (PMC8307932; doi:10.3390/cells10071792)

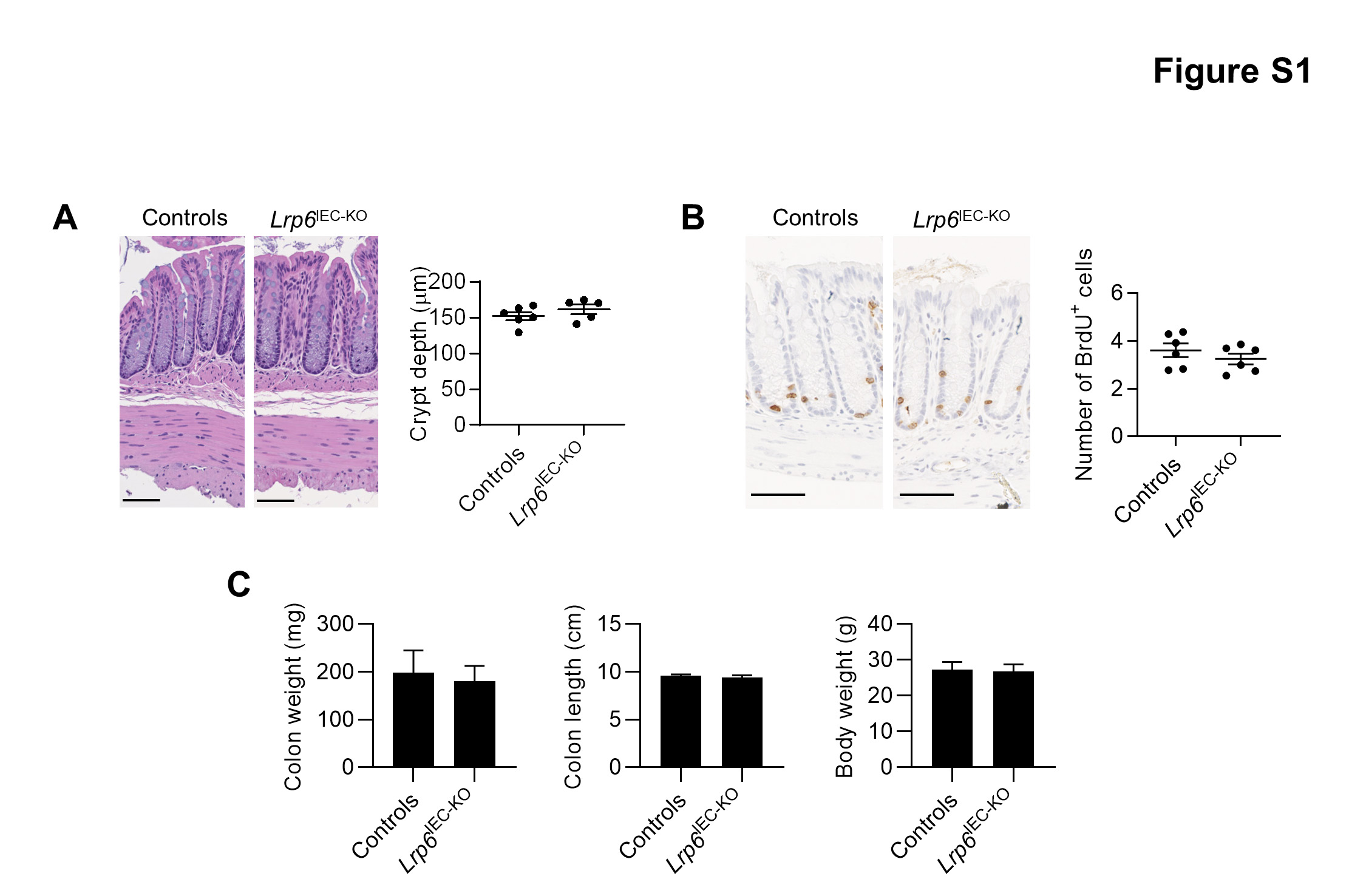

Supplement: Supplementary file 1 [file cells-10-01792-s001.zip › cells-1254428-supplementary/RaischFigureS1.jpg]

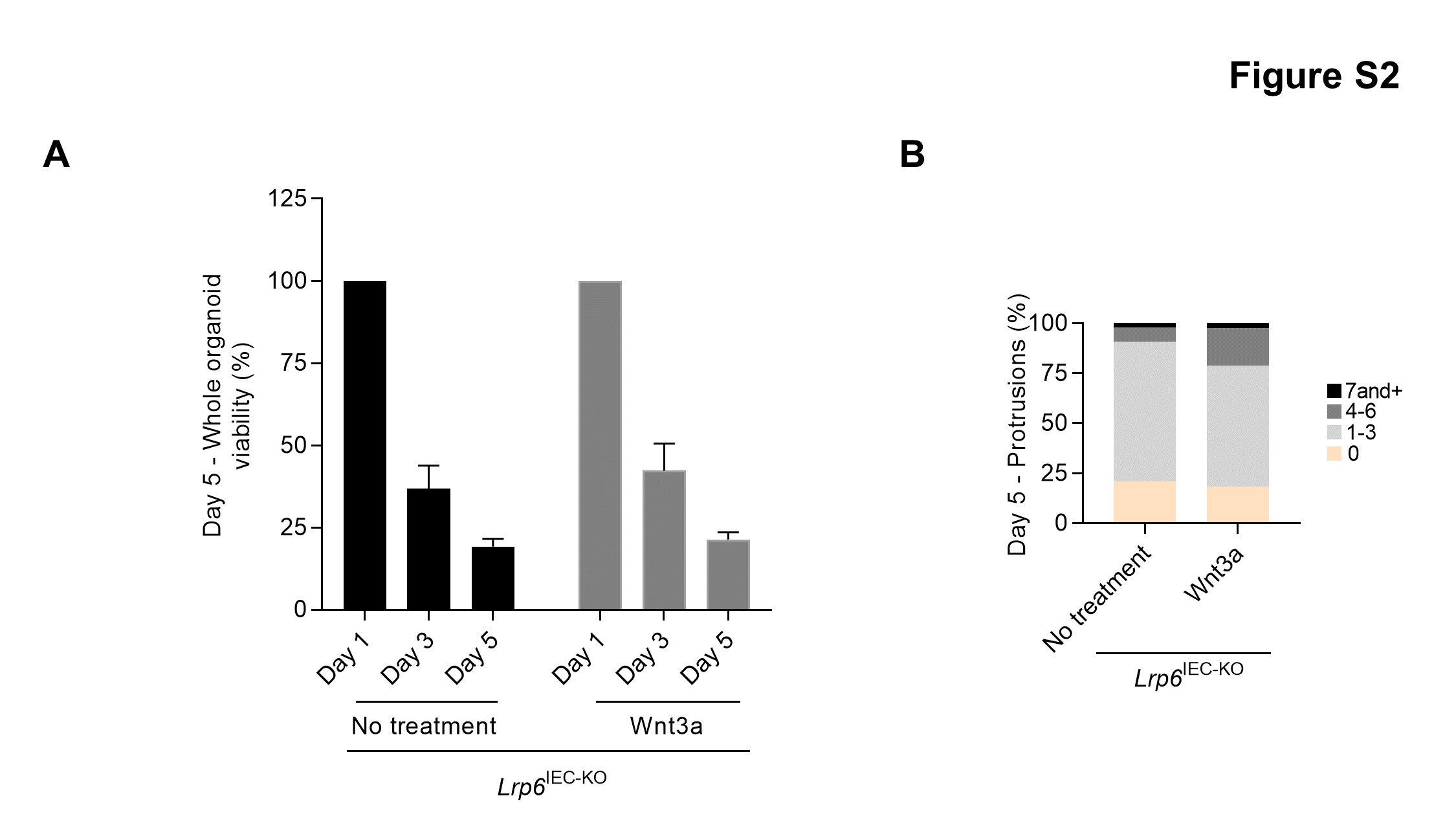

Supplement: Supplementary file 1 [file cells-10-01792-s001.zip › cells-1254428-supplementary/RaischFigureS2.jpg]

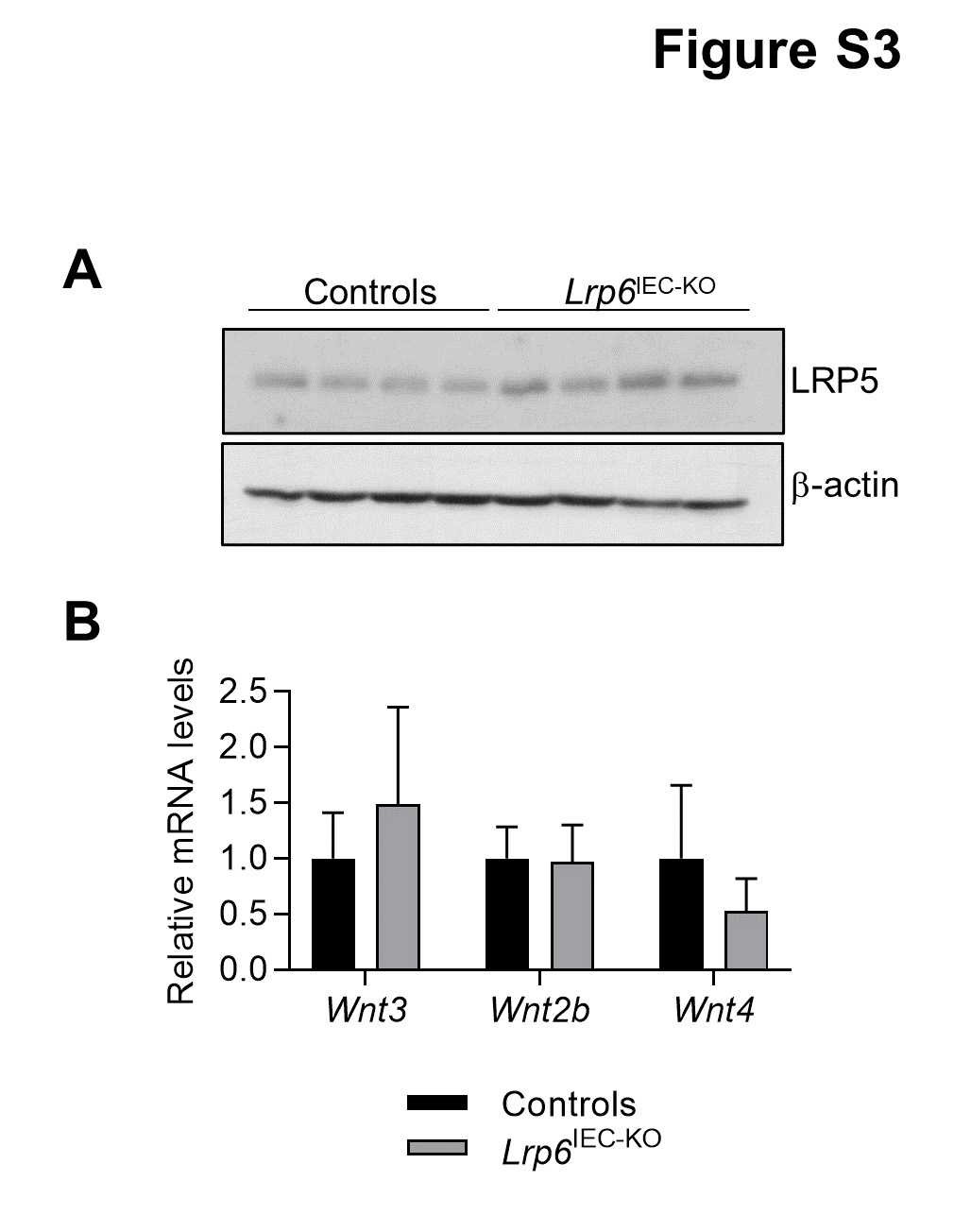

Supplement: Supplementary file 1 [file cells-10-01792-s001.zip › cells-1254428-supplementary/RaischFigureS3.jpg]
